# Survival from childhood cancer in northern England, 1968–2005

**DOI:** 10.1038/bjc.2011.341

**Published:** 2011-09-20

**Authors:** N O Basta, P W James, B Gomez-Pozo, A W Craft, R J Q McNally

**Affiliations:** 1 Institute of Health and Society, Newcastle University, Sir James Spence Institute, Royal Victoria Infirmary, Newcastle upon Tyne NE1 4LP, England, UK; 2 Northern Institute of Cancer Research, Newcastle University, Sir James Spence Institute, Royal Victoria Infirmary, Newcastle upon Tyne NE1 4LP, England, UK

**Keywords:** childhood cancer, survival, leukaemia, lymphoma, solid tumours

## Abstract

**Background:**

Cancer is the second most common cause of death in children in the developed world. The study investigated patterns and trends in survival from childhood cancer in patients from northern England diagnosed 1968–2005.

**Methods:**

Five-year survival was analysed using Kaplan–Meier estimation for four successive time periods. Cox regression analysis was used to explore associations with age and demographic factors.

**Results:**

The study included 2958 cases (1659 males and 1299 females). Five-year survival for all cancers improved significantly from 39% in 1968–1977 to 79% in 1998–2005 (*P*<0.001). Five-year survival for leukaemia increased from 24% to 81% (*P*<0.001), lymphoma from 46% to 87% (*P*<0.001), central nervous system tumours from 43% to 73% (*P*<0.001), bone tumours from 21% to 75% (*P*<0.001), soft tissue sarcoma from 30% to 58% (*P*<0.001) and germ cell tumours from 59% to 97% (*P*<0.001). Survival was worse for cases of acute lymphoblastic leukaemia (*P*<0.001) and astrocytoma (*P*<0.001) aged 10–14 years compared with 0–4-year olds.

**Conclusion:**

There were marked improvements in survival over a 38-year time span. Future work should examine factors that could influence further improvement in survival such as diagnosis delays.

Cancer is the second most common cause of death in children, aged 0–14 years, in the developed world. The continuous improvement in diagnostic and treatment strategies for cancer has led to significant improvements in survival for a wide range of childhood cancers (Kaatsch, 2010). Survival from childhood cancer in the United Kingdom has been included in international comparisons since the start of the European Cancer Registry-based Study on Survival and Care of Cancer Patients (EUROCARE) and the Automated Childhood Cancer Information System project (ACCIS) studies (Coebergh *et al*, 2001; Steliarova-Foucher *et al*, 2004; Pritchard-Jones *et al*, 2006; Gatta *et al*, 2009). Five-year survival for all European childhood cancers diagnosed during 1995–2002 was 81% (Gatta *et al*, 2009). Survival from childhood cancer in Great Britain (GB) improved substantially between 1971 and 1985 with 5-year survival reaching 76% for all cancer cases diagnosed during 1980–1991 (Stiller and Bunch, 1990; Stiller, 1994). A previous study from the Northern Region of England has examined survival in children and young adults diagnosed during the period 1968–1995 and reported significant improvements (Cotterill *et al*, 2000).

Population-based cancer registry data are regarded as highly reliable for comparison and analysis of survival (Pritchard-Jones and Stiller, 2007).

The aim of the present study was to investigate survival for cancer in children (aged 0–14 years) diagnosed during the period 1968–2005, and registered by the population-based Northern Region Young Persons’ Malignant Disease Registry (NRYPMDR). The study examined patterns and trends in survival, updating the previously published analysis and also presents separate diagnostic- and gender-specific results.

## Materials and methods

All cases aged 0–14 years diagnosed with a primary malignancy during the period 1968–2005 were obtained from the NRYPMDR. The NRYPMDR is a specialist registry, established in 1968, covering the counties of Northumberland, Tyne and Wear, Durham, Teesside and Cumbria (excluding Barrow-in-Furness). All cases of cancer in the region, diagnosed in 0–24-year olds, are notified to the registry. Cases are identified from multiple sources. Consultants throughout the region notify the registry of any malignancies in this age group. Data are periodically crosschecked with regional and national cancer registries. The registry has a high level of overall completeness and ascertainment, estimated to be >98%, with a very small proportion of cases lost to follow-up (<1% over the entire study period). Follow-up was achieved by regular checking of death certificates and hospital admission data (Cotterill *et al*, 2000). Previously registry data were grouped according to a modified version of the International Classification of Diseases for Oncology (ICDO-2; Kramárová *et al*, 1996) and modifications are documented by Cotterill *et al* (2000). The International Classification of Childhood Cancer third edition (ICCC-3) for coding morphology and primary site of diagnosis is now followed (Steliarova-Foucher *et al*, 2005). Following the ICCC third edition, benign and uncertain behaviour neoplasms of intracranial and intraspinal sites are included in both the central nervous system (CNS) tumour and the non-gonadal germ cell tumours groups. Myelodysplastic syndrome and other myeloprofilerative diseases are included under the subgroup ‘other leukaemia’.

### Statistical analysis

Survival at 5 years was analysed using Kaplan–Meier estimation, for each diagnostic group, within four successive subperiods 1968–1977, 1978–1987, 1988–1997 and 1998–2005 (Kaplan and Meier, 1958). Unadjusted trends in survival for each diagnostic group were assessed using log rank tests. The end point of interest was death from any cause, with date of diagnosis taken to be the time of origin. The NRYPMDR attempts to obtain comprehensive long-term follow-up on all childhood patients and was mostly complete until 31 December 2008.

Cox Proportional hazards regression analysis was used to model the probability of survival in relation to age at diagnosis (0–4, 5–9 and 10–14 years), gender, subperiods of diagnosis (1968–1977, 1978–1987, 1988–1997 and 1998–2005), area-level socioeconomic deprivation quintiles and area-level population density for all diagnostic groups and subgroups with >45 cases for the entire follow-up period. The significance of each covariate in the model was assessed using the partial likelihood ratio test. Non-nested models were compared using Akaike's Information Criterion (Collett, 2003). Hazard ratios (HRs) of variables were retained in the model only if they contributed significantly to the overall model fit. Simpler models, fitting subperiod of diagnosis and deprivation as continuous variables, were also assessed. The proportional hazards assumption was tested by examining Schoenfeld residuals and only those models that met the assumption were included in the results (Cox, 1972).

Cases were divided into five groups on the basis of the quintile of the distribution of Townsend deprivation score for the census ward of residence, from the most affluent to the most deprived (Townsend *et al*, 1988). Townsend scores were based on 1971, 1981, 1991 and 2001 censuses estimated for 2001 census ward geography (Norman *et al*, 2008; Norman, 2010). Population density for each electoral ward was calculated by dividing the population by the area. Wards were classified according to tertile of population density (for the period 1968–1985: low population density 2–1103 persons per km^2^, medium population density 1118–3290 persons per km^2^, high population density 3300–11 357 persons per km^2^; for the period 1986–1995: low 2–1052 persons per km^2^, medium 1058–3094 persons per km^2^, high 3113–10 680 persons per km^2^; and for the period 1996–2005: low 2–978 persons per km^2^, medium 981–2933 persons per km^2^, high 2974–8882 persons per km^2^). The ward population density figures for the periods 1968–1985, 1986–1995 and 1996–2005 were based, respectively, on the 1981, 1991 and 2001 censuses estimated for 2001 census ward geography. Statistical significance was taken to be *P*<0.05 in all analyses. Stata version 10 was used for the statistical analysis.

## Results

**Figure 1:**
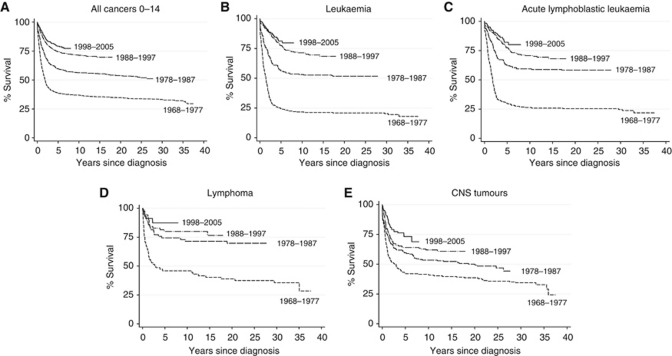
Kaplan–Meier survival plots. (**A**) All cancers 0–14, (**B**) leukaemia, (**C**) acute lymphoblastic leukaemia, (**D**) lymphoma, and (**E**) CNS tumours.

The study included a total of 2958 childhood cancer cases, diagnosed during the period 1968–2005 (1659 males and 1299 females). Five-year survival by period of diagnosis, for the diagnostic groups and subgroups is given in Table 1 and for males and females in Tables 2 and 3, respectively. Survival increased significantly over the study period (*P*<0.001) from a 5-year rate of 39% for the subperiod 1968–1977 to 60% for 1978–1987, 75% for 1988–1997 and 79% for 1998–2005 (Figure 1A). For all leukaemia and lymphoma combined survival increased from 29% to 60%, 78% and 83% for the four subperiods, respectively (*P*<0.001). Similarly, survival for solid tumours increased from 45% to 59%, 72% and 77%, respectively (*P*<0.001). Cox modelling showed there was little or no evidence of gender differences for survival among any of the specific diagnostic groups except for Hodgkin lymphoma (HL), where gender contributed significantly to the final model. Including subperiod of diagnosis, as a continuous variable, instead of a categorical variable, significantly improved the model fits for survival of several groups and subgroups of cancer (Table 1). It also showed that the apparent drop in survival for some diagnoses in the fourth time period compared with the third time period was not statistically significant. There was a steady increase in survival for ependymoma (*P*=0.02), osteosarcomas (*P*<0.001), soft tissue sarcoma (*P*<0.001) and rhabdomyosarcoma (*P*=0.001). There was also no evidence for an effect of area-level residential population density on survival so it was dropped from all final models.

### Leukaemia

There was a significant increase in survival for all leukaemias and specifically for acute lymphoblastic leukaemia (ALL; Figures 1B and C). Five-year survival for ALL increased from 30% in the first subperiod to 62%, 78% and 82% in the second, third and fourth subperiods, respectively (*P*<0.001). Cox regression modelling for ALL cases showed that age at diagnosis and categorical period of diagnosis were significant in the final model (*P*<0.001). After adjustment for period of diagnosis, the risk of death was higher for 10–14-year olds compared with those aged 0–9 years (HR=1.76; 95% CI=1.35–2.30). Survival for acute non-lymphocytic leukaemia (ANLL) was very low in the earlier half of the study but improved markedly during the latter half with a significantly large increase from 14% in 1978–1987 to 78% in 1988–1997 and 77% in 1998–2005 (*P*<0.001).

### Lymphoma

**Figure 2:**
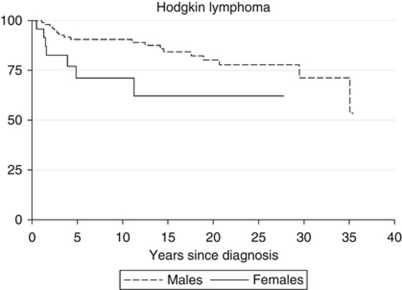
Kaplan–Meier survival plot by gender.

Survival improved significantly over the study period for all lymphomas (*P*<0.001; Figure 1D). Survival for HL improved over the study period (*P*=0.003) from a high level of 73% in the first subperiod to 90% in the second subperiod but with little subsequent improvement. Cox regression modelling for HL cases showed that gender and subperiod of diagnosis as a continuous variable were significant in the final model. After adjustment for period of diagnosis, girls had a higher risk of death compared with boys (HR=3.43; 95% CI=1.37; Figure 2). There was a marked improvement in 5-year survival for non-Hodgkin lymphoma (NHL) from 23% to 83% in the fourth subperiod (*P*<0.001), as shown by the Cox analysis (*P* for linearity <0.001).

### CNS tumours

Survival improved significantly over the study period for all cases of CNS tumours combined, with 5-year survival improving from 43% to 73% (*P*<0.001; Figure 1E). There were significant improvements in survival for ependymoma from 39% to 69% (*P*=0.013) and for astrocytoma from 60% to 77% (*P*=0.049), but the apparent improvement for primitive neuroectodermal tumours (PNETs) from 24% to 63% was not significant (*P*=0.236).

For cases of PNET, Cox regression modelling showed that age at diagnosis and quintile of deprivation as a categorical variable were significant in the final model, but subperiod of diagnosis was not significant. Survival was significantly better in the areas with highest deprivation (HR=0.10; 95% CI=0.03–0.32), and better for older children aged 5–9 compared with those aged 0–4 years (HR=0.33; 95% CI=0.18–0.61) and also better for children aged 10–14 years although this was not significant (HR=0.58; 95% CI=0.31–1.08).

For ependymoma cases, the final model included subperiod of diagnosis and deprivation as continuous variables. Survival for ependymoma cases was better as deprivation got worse with HR decreasing by a factor of 0.77 (95% CI=0.62–0.97) for each quintile of deprivation.

For astrocytoma cases, the final model included age at diagnosis, subperiod of diagnosis as a continuous variable and quintile of deprivation as a categorical variable. Survival for astrocytoma cases was better in the areas with the highest deprivation (HR=0.36; 95% CI=0.16–0.80). However, survival was significantly poorer for children aged 10–14 years (HR=2.51; 95% CI=1.37–4.62).

### Peripheral nervous system tumours

There was a significant increase in overall survival for neuroblastoma from 13% to 65% (*P*<0.001).

### Retinoblastoma

Survival was consistently high and between 86 and 100% with no significant variation (*P*=0.236).

### Renal tumours

There was a significant increase in the overall survival for Wilms tumour (*P*=0.029).

### Bone tumours

Overall survival for bone tumours significantly increased from 21% to 75% (*P*<0.001). Cox regression showed that the improvement in survival for both osteosarcoma and Ewing Sarcoma was steady (*P*<0.001) and the apparent drop from 71% in the third period to 69% in the fourth period was most likely due to random sampling variation due to small numbers.

### Soft tissue sarcomas

Survival significantly increased for all soft tissue sarcoma from 30% to 58% (*P*=0.001) and specifically for rhabdomyosarcoma from 19% to 59% (*P*=0.003). Cox regression showed that the improvement in survival for rhabdomyosarcoma was steady (*P*=0.001) and the apparent drop from 62% in the third period to 59% in the fourth period was most likely due to random sampling variation.

### Germ cell tumours

Five-year survival for all gonadal germ cell tumours significantly improved from 70% to 100% (*P*=0.023). For all non-gonadal germ cell tumours, there was a significant increase in 5-year survival from 43% to 95% (*P*=0.007).

### Carcinomas

The apparent increase in survival for carcinomas was not statistically significant (*P*=0.273).

## Discussion

This study provides up-to-date estimates of childhood cancer survival from a population-based cancer registry in northern England. The population of the Northern Region is ethnically homogenous with fewer than 2% from ethnic minorities (Office of Population Censuses and Surveys Census Division, General Register Office (Scotland) Census Branch, 1983; Office for National Statistics, 1991, 2001). The incidence of childhood cancer in this area is similar to England in general (Magnanti *et al*, 2008a, 2008b). Five-year survival for all cancer in children (aged 0–14 years) have improved considerably over the study period. The largest improvements in survival in more recent years were seen for ANLL (from the third subperiod) and for NHL and sympathetic nervous system tumours (especially in the last subperiod). However, the new data showed continued improvements in survival for the groups comprising all cancers, all haematological malignancies and all solid tumours since the previously published report (Cotterill *et al*, 2000). The previously published study only reported survival for all cancers, haematological malignancies and solid tumours collectively; in the present study, we report both diagnostic- and gender-specific survival.

The improvement in overall cancer survival during the study period is consistent with other studies. The overall 5-year survival from cancer was similar to a previous report from the whole of the United Kingdom where the 5-year survival for the period 1991–2000 was 75% (Stiller, 2007). Our findings were also similar to those reported by other studies, including the EUROCARE study (5-year survival for all childhood cancers combined, diagnosed 1995–2002: 81% Gatta *et al*, 2009), the Surveillance, Epidemiology and End Results (SEERs) programme in the United States (cases diagnosed 1999–2006: 79.9% Altekruse *et al*, 2010) and from Australia (cases diagnosed 1997–2006: 79.5% Baade *et al*, 2010). For specific diagnostic subgroups, similar findings were also reported. Childhood cancer survival figures reported from the SEER and the Australian studies were produced using relative survival. However, the results from the EUROCARE study were observed survival. Using relative survival makes little difference to the estimates because deaths due to other causes are rare in children (Gatta *et al*, 2009).

In GB, the 5-year survival for ALL, HL and NHL diagnosed during 1991–2000 was 87%, 95% and 79%, respectively (Stiller, 2007); and for all European children, diagnosed in 1995–2002 were 85%, 95% and 82% (Gatta *et al*, 2009). Since the greatest improvements in survival for leukaemia in early 1990s there have been very little subsequent improvements.

The 5-year survival for ANLL has seen great improvements for children diagnosed 1987–2005 (77%) and the reason for this improvement was likely to be due to additional more intensive therapy protocols that were introduced by the United Kingdom Medical Research Council (UK MRC) trials in early 1980s (Gibson *et al*, 2005). These protocols have been followed in the treatment centres in the northern region. The 5-year overall survival for children with ANLL who were treated on UK MRC trials between 1988 and 2002 was 66% (Gibson *et al*, 2005) and for ANLL cases diagnosed 1996–2000 in GB was 65% (Stiller, 2007).

The overall 5-year survival results for CNS tumours showed significant improvements and there were non-significant improvements for PNETs. CNS tumours are a very heterogeneous diagnostic group. Five-year survival from the United Kingdom, for cases diagnosed during 1991–2000 was 69%, 66% and 79% for all CNS tumours, ependymoma and astrocytoma, respectively (Stiller, 2007). Five-year survival for astrocytoma diagnosed 1995–2005 reported for Europe (78%) (Gatta *et al*, 2009) was similar to the present study (77%), but the 5-year survival for astrocytoma diagnosed during 1999–2006 reported in the United States by the SEER programme (Altekruse *et al*, 2010) was higher (84.8%), although the same ICCC-3 classification of coding morphology was followed by the three studies (Steliarova-Foucher *et al*, 2005). Survival for PNETs reported in the current study (63%) is similar to that reported from the EUROCARE study for embryonal CNS tumours (which were mainly medulloblastoma and PNET) diagnosed during 1995–2002 (66%) (Gatta *et al*, 2009).

For peripheral nervous system tumours, which comprise mainly neuroblastoma, we found recent marked increases in the 5-year survival especially in the last subperiod. An increase in survival for neuroblastoma cases diagnosed in the United Kingdom was persistent over the time period, with the greatest increases in survival from 1971–1975 to 1981–1985 with little change in 1986–1990 but then followed by a further increase in 1996–2000 (Stiller, 2007). Our results show that the 5-year survival for neuroblastoma in the last subperiod was 65%, which is higher than the 5-year survival reported from the whole of United Kingdom, for cases diagnosed 1991–2000 (59%) (Stiller, 2007), but lower than reported from Europe (72%) (Gatta *et al*, 2009) and the United States (73%) (Altekruse *et al*, 2010).

The overall 5-year survival for bone tumours diagnosed during 1998–2005 (75% osteosarcomas: 69% Ewing tumours: 73%) was higher than that reported from the earlier study from Northern England and the West Midlands for cases diagnosed 1981–2000 where overall 5-year survival for the period 1995–2000 was 62% (osteosarcomas: 57% and Ewing sarcoma: 70% Eyre *et al*, 2009).

Soft tissue sarcoma had the lowest 5-year survival of all diagnostic groups (58%) which is lower than rates reported from the United Kingdom for cases diagnosed 1991–2000 (66% Stiller, 2007). Germ cell tumours had the highest 5-year survival and this was similar to previous reports from the United Kingdom (Stiller, 2007) and from Europe (Gatta *et al*, 2009).

The Cox regression analyses showed that survival for ALL and astrocytoma was influenced by age of the patients, with older children faring worse. Our data are consistent with findings from the whole of the United Kingdom for ALL and astrocytoma where older children aged 5–14 years had worse prognosis than children aged 1–4 years (Stiller, 2007). The age at which children are diagnosed with cancer is an important prognostic factor for several childhood cancers, which may be used in several classifications of high risk patients and stratification for treatment (Coebergh *et al*, 2001). Children diagnosed with ALL, who were patients in the UK MRC clinical trials protocols between 1980 and 2001, and who were older than 10 years were consistently associated with high risk disease and worse outcome compared with children younger than 10 years (Mitchell *et al*, 2010). Also, the delay in access to care among adolescents diagnosed with cancer may contribute to worse survival for older children (Albritton and Eden, 2008). Survival from ALL is reported to be worse in adolescent groups compared with younger patients (Feltbower *et al*, 2009).

There were no significant differences in survival for any of the cancer types by area-level socioeconomic deprivation except for CNS tumours where survival was better for children resident in more deprived areas compared with those from more affluent areas. A similar unexpected association between poorer survival and higher affluence was previously reported for children and young adults diagnosed with CNS tumours in Yorkshire (Feltbower *et al*, 2004). These findings were in contrast to the data from England and Wales. Coleman *et al* (1999) have shown that deprivation has a strong influence on cancer survival rates in adults, but much less influence in children. The findings of the present study may reflect the fact that more deprived areas are mostly constituted of urban areas which could have closer geographic access to diagnostic and treatment services. However, these findings should be interpreted with caution and the role of chance cannot be ruled out. We found little evidence of difference in survival between the sexes, apart for HL which was based on a small number of girls. Previous studies have found evidence of sex-specific differences in survival for ALL (Coebergh *et al*, 2001; Stiller, 2007; Johnston *et al*, 2010). Johnston *et al* (2010) also report less favourable outcomes for neuroblastoma in boys than in girls .

In conclusion, our results indicate that there has been substantial improvement in survival for childhood cancer in the North of England over the last four decades. This is resulting in a growing population of long-term survivors who need long-term follow-up and catering for their needs to minimise morbidity, prevent secondary cancer and to normalise their lives. Improvements in survival may generally be attributed to a number of changes in the management and treatment of childhood cancers. Future work should analyse geographical patterning in cancer survival and other factors that may lead to delays in diagnosis (Richards, 2009).

## Figures and Tables

**Table 1:** Percentage 5-year survival by time period and diagnostic group

		**1968–1977**		**1978–1987**		**1988–1997**		**1998–2005**			
**Diagnostic group**	***N***	**% Survival**	**95% CI**	**% Survival**	**95% CI**	**% Survival**	**95% CI**	**% Survival**	**95% CI**	***P* for trend**	***P* for linearity**
*Leukaemia*	895	24	19–30	56	49–62	77	72–82	81	73–87	<0.001	—
ALL	742	30	24–36	62	55–68	78	71–83	82	73–88	<0.001	—
ANLL	128	2	0–10	14	5–30	78	55–90	77	55–89	<0.001	—
											
*Lymphoma*	289	46	34–57	74	62–83	80	69–88	87	76–94	<0.001	<0.001
Hodgkin	128	73	54–85	90	73–97	96	77–99	93	73–98	0.003	0.003
Non-Hodgkin	134	23	11–37	62	45–75	65	46–78	83	55–94	<0.001	<0.001
											
*CNS*	702	43	35–50	60	52–66	64	56–71	73	64–80	<0.001	<0.001
Ependymoma	72	39	17–60	26	11–45	75	41–91	69	34–88	0.013	0.02
Astrocytoma	264	60	46–72	79	65–88	72	59–81	77	63–87	0.049	0.001
PNET	124	24	11–41	45	27–61	43	23–62	63	39–80	0.236	—
											
*Peripheral nervous system*	197	17	7–29	32	20–45	52	38–63	66	45–80	<0.001	<0.001
Neuroblastoma	187	13	5–25	33	20–46	51	37–63	65	45–80	<0.001	<0.001
											
*Retinoblastoma*	88	92	76–97	86	54–96	96	75–99	100	—	0.236	—
											
*Renal*	159	63	48–75	76	58–87	83	69–91	90	66–98	0.029	0.004
Wilms tumour	157	63	47–74	76	58–87	83	68–91	90	66–98	0.029	0.004
											
*Bone*	126	21	10–34	34	19–51	71	51–85	75	50–89	<0.001	<0.001
Osteosarcoma	74	17	6–33	50	26–70	71	43–87	69	30–89	<0.001	<0.001
Ewing sarcoma	46	25	6–50	14	2–37	73	37–90	73	28–93	0.003	0.002
											
*Soft tissue sarcoma*	202	30	19–42	53	38–66	69	55–80	58	36–75	<0.001	<0.001
Rhabdomyosarcoma	114	19	8–35	50	32–66	62	40–77	59	33–77	0.003	0.001
											
*Germ cell tumour*	115	59	33–78	86	62–95	94	80–99	97	81–100	<0.001	<0.001
Gonadal	50	70	33–89	88	59–97	100	—	100	—	0.023	0.023
Non-gonadal	65	43	10–73	80	20–97	92	72–98	95	72–99	0.007	0.004
											
Carcinomas	93	65	40–82	88	59–97	89	72–96	76	47–90	0.273	—
											
Total leukaemia and lymphoma	1184	29	24–34	60	55–66	78	73–82	83	77–88	<0.001	—
Total solid tumours	1774	45	41–50	59	54–64	72	68–76	77	72–81	<0.001	—
Total all cancers^a^	2958	39	35–42	60	56–63	75	71–77	79	75–83	<0.001	—

Abbreviations: CI=confidence intervals; ALL=acute lymphoblastic leukaemia; ANLL=acute non-lymphocytic leukaemia; CNS=central nervous system; PNET=primitive neuroectodermal tumour.

Table shows all diagnostic groups with >45 patients diagnosed over the study period.

a Total including other miscellaneous groups.

**Table 2:** Percentage 5-year survival by time period and diagnostic group for males

		**1968–1977**		**1978–1987**		**1988–1997**		**1998–2005**		
**Diagnostic group**	***N***	**% Survival**	**95% CI**	**% Survival**	**95% CI**	**% Survival**	**95% CI**	**% Survival**	**95% CI**	***P* for trend**
*Leukaemia*	509	24	17–31	53	44–61	74	66–81	81	70–89	<0.001
ALL	428	28	21–37	59	49–68	74	65–81	82	69–90	<0.001
ANLL	70	4	0–17	21	7–41	85	51–96	72	34–90	<0.001
										
*Lymphoma*	215	53	39–65	70	56–80	86	73–93	92	76–97	<0.001
Hodgkin	103	82	62–92	88	67–96	100		94	67–99	0.095
Non-Hodgkin	93	24	11–41	56	38–71	68	43–84	89	43–98	<0.001
										
*CNS*	386	41	31–51	58	48–66	63	52–72	74	61–83	<0.001
Ependymoma	51	50	21–74	17	4–37	63	23–86	90	47–99	0.005
Astrocytoma	137	57	37–73	81	62–91	68	49–81	74	49–88	0.158
PNET	78	26	10–47	35	16–55	40	16–63	58	31–78	0.574
										
*Sympathetic nervous system*	102	13	3–30	25	11–42	47	28–63	53	26–75	0.002
Neuroblastoma	97	10	2–26	26	11–43	46	28–63	53	26–75	<0.001
										
*Renal*	71	57	34–74	82	45–95	85	64–94	72	24–93	0.140
Wilms tumour	70	57	34–74	82	45–95	84	63–94	72	24–93	0.160
										
*Bone*	67	29	13–48	23	6–47	60	32–80	92	57–99	<0.001
										
*Soft tissue sarcoma*	130	29	15–45	57	39–72	71	53–83	61	36–78	<0.001
Rhabdomyosarcoma	75	24	7–45	52	31–70	63	35–81	58	28–79	0.071
										
Germ cell tumour	55	67	34–86	93	59–99	100	—	93	59–99	0.016
										
Total leukaemia and lymphoma	724	32	26–38	58	51–65	77	71–83	85	76–90	<0.001
Total solid tumours	935	43	37–49	57	50–63	71	64–76	75	67–81	<0.001
Total all cancers	1659	38	33–42	58	53–62	74	69–78	79	73–83	<0.001

Abbreviations: CI=confidence intervals; ALL=acute lymphoblastic leukaemia; ANLL=acute non-lymphocytic leukaemia; CNS=central nervous system; PNET=primitive neuroectodermal tumour.

Table shows all diagnostic groups with >45 patients diagnosed over the study period.

**Table 3:** Percentage 5-year survival by time period and diagnostic group for females

		**1968–1977**		**1978–1987**		**1988–1997**		**1998–2005**		
**Diagnostic group**	***N***	**% Survival**	**95% CI**	**% Survival**	**95% CI**	**% Survival**	**95% CI**	**% Survival**	**95% CI**	***P* for trend**
*Leukaemia*	386	25	17–33	60	50–69	82	73–89	81	68–89	<0.001
ALL	314	31	22–41	65	54–74	84	73–90	82	65–91	<0.001
ANLL	58	0	—	0	—	70	33–89	80	49–93	<0.001
										
Lymphoma	74	20	5–42	92	57–99	65	40–82	79	57–91	<0.001
										
*CNS*	316	44	33–54	63	50–73	65	53–75	73	59–82	<0.001
Astrocytoma	127	64	42–79	76	52–89	77	57–88	80	63–90	0.300
PNET	46	20	3–47	64	30–85	50	15–77	78	36–94	0.413
										
*Sympathetic nervous system*	95	21	7–41	41	21–60	57	37–72	76	45–91	<0.001
Neuroblastoma	90	17	4–37	41	21–60	56	35–72	75	44–91	<0.001
										
Retinoblastoma	50	87	65–96	86	33–98	100	—	100	—	0.433
										
Renal	88	69	48–83	74	51–87	81	57–92	100	—	0.153
Wilms tumour	87	68	46–83	74	51–87	81	57–92	100	—	0.137
										
Bone	59	11	2–28	42	20–62	85	51–96	47	12–76	0.001
										
Soft tissue Sarcoma	72	30	14–49	43	18–66	65	40–82	58	23–82	0.099
										
Germ cell tumour	60	40	5–75	71	26–92	91	69–98	100	—	<0.001
										
Carcinomas	54	69	37–87	91	51–99	86	62–95	76	33–94	0.500
										
Total leukaemia and lymphoma	460	24	17–32	63	54–72	79	70–86	81	70–88	<0.001
Total solid tumours	839	48	41–54	61	54–68	74	68–79	79	72–85	<0.001
Total all cancers	1299	39	34–44	62	56–67	76	71–80	80	74–84	<0.001

Abbreviations: CI=confidence intervals; ALL=acute lymphoblastic leukaemia; ANLL=acute non-lymphocytic leukaemia; CNS=central nervous system; PNET=primitive neuroectodermal tumour.

Table shows all diagnostic groups with >45 patients diagnosed over the study period.
